# Coexistence of inhibitory and activating killer-cell immunoglobulin-like receptors to the same cognate HLA-C2 and Bw4 ligands confer breast cancer risk

**DOI:** 10.1038/s41598-021-86964-y

**Published:** 2021-04-12

**Authors:** Elham Ashouri, Karan Rajalingam, Shaghik Barani, Shirin Farjadian, Abbas Ghaderi, Raja Rajalingam

**Affiliations:** 1 UCLA Immunogenetics Center, David Geffen School of Medicine at UCLA, University of California, Los Angeles, CA 90095 USA; 2 Shiraz Institute for Cancer Research, School of Medicine, Shiraz University of Medical Sciences, Shiraz, Iran; 3 Department of Immunology, School of Medicine, Shiraz University of Medical Sciences, Shiraz, Iran; 4 Immunogenetics and Transplantation Laboratory, Department of Surgery, University of California San Francisco, San Francisco, CA USA

**Keywords:** Cancer, Immunology, Medical research, Oncology, Risk factors

## Abstract

Human leukocyte antigen (HLA) class I-specific killer-cell immunoglobulin-like receptors (KIR) regulate natural killer (NK) cell function in eliminating malignancy. Breast cancer (BC) patients exhibit reduced NK-cytotoxicity in peripheral blood. To test the hypothesis that certain *KIR-HLA* combinations impairing NK-cytotoxicity predispose to BC risk, we analyzed *KIR* and *HLA* polymorphisms in 162 women with BC and 278 controls. *KIR-Bx* genotypes increased significantly in BC than controls (83.3% vs. 71.9%, OR 1.95), and the increase was more pronounced in advanced-cancer (OR 5.3). No difference was observed with inhibitory *KIR* (*iKIR*) and *HLA-*ligand combinations. The activating *KIR* (*aKIR) and HLA*-ligand combinations, *2DS1* + *C2* (OR 2.98) and *3DS1* + *Bw4* (OR 2.6), were significantly increased in advanced-BC. All patients with advanced-cancer carrying *2DS1* + *C2* or *3DS1* + *Bw4* also have their *iKIR* counterparts *2DL1* and *3DL1*, respectively. Contrarily, the *2DL1* + *C2* and *3DL1* + *Bw4* pairs without their *aKIR* counterparts are significantly higher in controls. These data suggest that NK cells expressing iKIR to the cognate HLA-ligands in the absence of putative aKIR counterpart are instrumental in antitumor response. These data provide a new framework for improving the utility of genetic risk scores for individualized surveillance.

## Introduction

Breast cancer is the most commonly diagnosed cancer and the leading cause of cancer-related deaths in women worldwide^1,2^. Both innate and adaptive immune systems play a central role in preventing primary and recurrence of breast cancer^3^. Natural killer (NK) cells, a subset of innate lymphoid cells representing 5–20% of peripheral blood mononuclear cells, mediate a fast-acting first-line defense against tumor transformation and viral infection^4^. NK cells can lyse target cells quickly by direct cytotoxicity in an antigen-independent manner without the “priming” period required by T cells^5^. NK cells also produce high levels of interferon-γ (IFN-γ) and a wide range of pro-inflammatory cytokines and chemokines, which contribute to the shaping of adaptive immune responses^6^. The direct involvement of NK cells in controlling growth and metastasis of breast cancer was demonstrated by using T, B, and NK knock-out NOG mice and T and B knock-out NOD/SCID mice^7^. A high level of natural cytotoxic activity of peripheral-blood lymphocytes was associated with reduced cancer risk^8^. Women with breast cancer exhibit significantly reduced NK cell cytotoxicity in peripheral blood compared to healthy individuals^9,10^.

**Figure 1:**
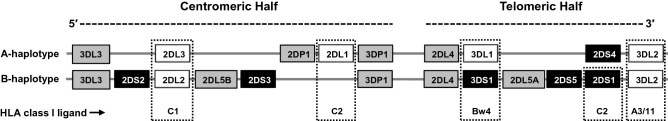
Map of group A and B KIR haplotypes. Distinct KIR haplotypes carry quantitively and qualitatively contrasting KIR gene content. Inhibitory KIR genes are depicted in white boxes. Activating KIRs are shown in dark boxes. Gray boxes represent pseudogenes or KIR genes with unclear function. HLA class I ligands for specific KIR are identified in dotted boxes. The centromeric and telomeric half are marked.

In contrast to other innate immune cells, NK cell population is highly heterogeneous and uses many specific germline-encoded repertoires of activating and inhibitory receptors to recognize target cells^11^. The KIR receptors are considered the key receptors that control human NK cell development and effector function^12^. Fourteen KIRs triggering either inhibition (3DL1-3, 2DL1-3, 2DL5) or activation (3DS1, 2DS1-5), or both (2DL4) have been identified. The *KIR* gene family displays a high degree of diversity determined by the variability in *KIR* gene content between haplotypes and allelic polymorphism of each gene^13,14^ (Fig. 1). Based on gene content, the *KIR* haplotypes are broadly classified into two groups: *A* and *B*^15^. Both *A* and *B* groups of haplotypes contain all four framework genes (*KIR3DL3-3DP1-2DL4-3DL2*) but differ substantially by the quantity and the quality of other KIR gene content. In addition to framework genes, group *A* haplotypes have a fixed set of four genes (*KIR2DL3-2DL1-3DL1-2DS4*) and encode inhibitory KIRs, 2DL1, 2DL3, 3DL1, and 3DL2, specific for all four HLA class I ligands, C2, C1, Bw4, and A3/A11 respectively. Group *B* haplotypes have variable gene content and comprising one or more of the seven genes that are not part of the *A* haplotype (*2DL2, 2DL5, 2DS1, 2DS2, 2DS3, 2DS5, 3DS1*). While group *A* haplotypes contain only *KIR2DS4* as an activating gene, group *B* haplotypes contain up to five activating *KIRs*—*KIR2DS1, 2DS2, 2DS3, 2DS5*, and *3DS1*.

By interacting with specific self-HLA class I ligands, the inhibitory KIR receptors set the threshold for NK cell function, a maturation programming in NK cells termed licensing or education^16,17^. The licensing render the subsequent ability to survey, recognize and kill stressed target cells that have lost HLA class I molecules due to tumor transformation or viral infection. In the absence of inhibitory KIR-HLA interactions, NK cells become hyporesponsive or anergic. The ligand specificity for the activating KIRs remains elusive. Certain activating KIRs display a high degree of sequence homology with the corresponding inhibitory KIR in their extracellular Ig domains. Therefore these activating KIRs are expected to exhibit a binding specificity similar to their inhibitory counterpart. For example, KIR2DS1 and 2DL1 differ by only seven amino acids in their extracellular portion, and therefore, KIR2DS1 is known to bind weakly to HLA-C2^18–20^. The KIR3DS1 that shares the highest sequence homologies with 3DL1 in their extracellular portion is shown to bind to HLA-Bw4 in a peptide-dependent manner or with the existence of a particular KIR-HLA combinations^21,22^. The KIR2DL2 and 2DL3 bind to HLA-C1, but KIR2DS2, whose extracellular domain differs from KIR2DL2 and 2DL3 by only 3 and 4 amino acids, respectively, binds to HLA-A*11:01 complexed with a vaccinia viral peptide^23^. Activating receptor KIR2DS4 recognizes some C1- or C2-bearing HLA-C allotypes, as well as the HLA-A3/11 epitope^24,25^. Using KIR-Fc fusion protein on a panel of 97 distinct HLA class I molecule-coated microbeads, specific alleles of the activating KIR2DS5 were shown to bind HLA-C2 allotypes^26^. The activating KIR2DS3 was not demonstrated to bind any HLA^24,25^.

Given that *KIR* genes at chromosome 19 and *HLA* genes at chromosome 6 are polymorphic and display significant variations, the independent segregation of these unlinked gene families produces extraordinary diversity in the number and type of *KIR-HLA* pairs inherited in individuals^27,28^. *KIR-HLA* variation affects the KIR repertoire of NK cell clones, NK cell maturation, the capability to deliver signals, and the NK cell response to human diseases^29^. To test the hypothesis that certain *KIR-HLA* combinations that impair NK cell cytotoxicity predispose to breast cancer risk, we analyzed a well-defined cohort of breast cancer patients and healthy controls from the native population of southern Iran.

## Results

### *Bx KIR* genotypes are positively associated with breast cancer

**Figure 2:**
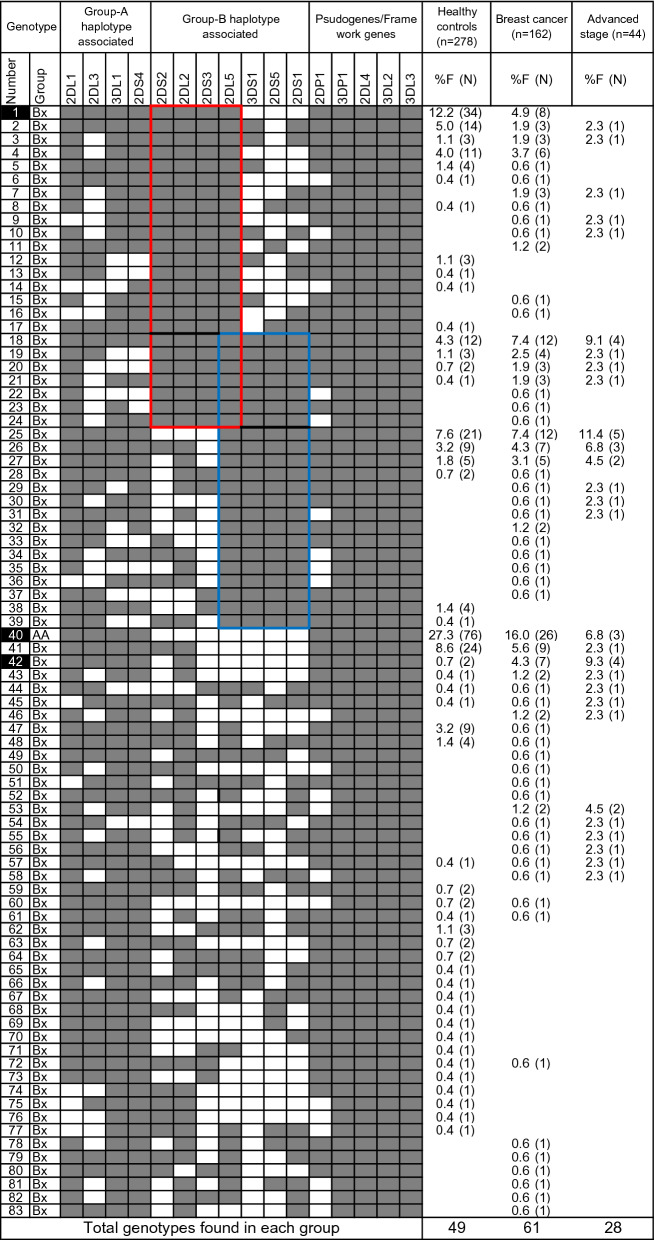
*KIR* gene content diversity in patients with breast cancer. Eighty-three distinct *KIR* genotypes were observed that differ from each other by the presence (shaded box) or absence (white box) of 16 KIR genes. The frequency (%F) of each genotype is expressed as a percentage and defined as the number of individuals having that specific genotype (N) divided by the number of individuals studied (n) in each group. The frequency of genotypes # 1, #40, and #42 was significantly different between patients and controls and are marked by dark boxes. The C4 and T4 linkage groups are marked by red and blue boxes, respectively.

To test the possibility that *KIR* genes are involved in the risk of breast cancer, we determined the presence and absence of all 16 *KIR* genes and cognate *HLA* class I ligands in 162 patients with breast cancer and 278 healthy controls from the native population of southern Iran. A panel of 83 genotypes differing by *KIR* gene content were identified in this cohort of 440 native Iranians (Fig. 2). Sixty-one *KIR* genotypes were encountered in patients with breast cancer, while only 49 *KIR* genotypes were found in controls. Thirty-four *KIR* genotypes (41%) occurred exclusively in patients with breast cancer. Only three genotypes (#1, #40, and #42) occurred at significantly different frequencies between patients and controls. Two of them occurred at significantly low frequencies in patients compared to controls; *AA* genotype (genotype#40: 16.0% vs. 27.3%, *p* = 0.007, Odd ratio (OR) = 0.51, 95% confidence interval (CI) = 0.31–0.83) and the most common *Bx* genotype (genotype#1: 4.9% vs. 12.2%, *p* = 0.015, OR = 0.37, CI = 0.17–0.83) (Fig. 2). The decrease of genotypes#40 and #1 was more prominent in patients with the advanced stage of cancer (genotype#40: 6.8% vs. 27.3%, *p* = 0.007, OR = 0.19, CI = 0.06–0.65; genotype#1: 0% vs. 12.2%, *p* = 0.07, OR = 0.08, CI = 0.005–1.32). Genotype#42 occurred at significantly higher frequency in patients than controls (4.3% vs. 0.7%, *p* = 0.02, OR = 6.23, CI = 1.28–30.4), and the increase is pronounced in patients with the advanced stage of cancer (9.3% vs. 0.7%, *p* = 0.003, OR = 13.8, CI = 2.45–77.8).

**Figure 3:**
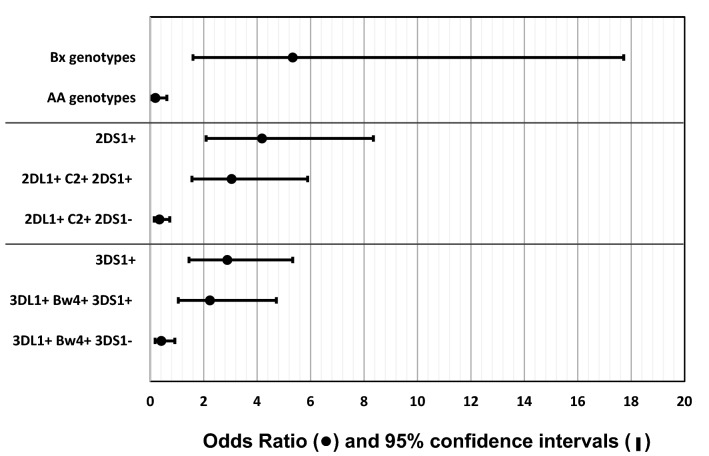
Selected KIR and HLA association with advanced-stage breast cancer. Forest plot depicting odds ratios (circles) with 95% confidence intervals for the association between selected KIR and HLA genotypes and advanced-stage breast cancer compared to healthy controls.

The *Bx* genotypes comprising 2–6 activating *KIR* genes, occurred more frequently in patients compared to controls (83.3% vs. 71.9%, *p* = 0.007, OR = 1.95, CI = 1.19–3.18) (Table 1). The increase of *Bx* genotypes was prominent in patients with advanced stage of cancer (93.2% vs. 71.9%, *p* = 0.003, OR = 5.33, CI = 1.6–17.72) (Table 1, Fig. 3). Particularly, the *Bx* genotypes that carry telomeric *KIR3DS1-2DL5-2DS5-2DS1* gene cluster (i.e., T4 linkage group) occurred at increased frequency in patients with breast cancer compared to healthy controls (37% vs. 21.6%, *p* = 0.0005, OR = 2.14, CI = 1.39–3.28), and the increase was further pronounced in patients with advanced stage cancer (45.5% vs. 21.6%, *p* = 0.001, OR = 3.03, CI = 1.57–5.85). Contrariwise, the *AA* genotype decreased significantly in breast cancer patients compared to controls (16.7% vs. 28.1%, *p* = 0.007, OR = 0.51, CI = 0.31–0.84); the decrease was pronounced in patients with advanced stage breast cancer (6.8% vs. 28.1%, *p* = 0.003, OR = 0.19, CI = 0.06–0.62) (Table 1, Fig. 3).

**Table 1:** Frequency of *KIR* genotypes, linked gene groups, and genes in breast cancer patients and controls.

*KIR*	Healthy controls		Breast cancer (BC)						Comparison			
			BC		Early		Advanced		BC vs Controls		Advanced vs Controls	
	n = 278		n = 162		n = 70		n = 44		*p* value	OR (95%CI)	*p* value	OR (95%CI)
*KIR* genotypes	%F	(N +)	%F	(N +)	%F	(N +)	%F	(N +)				
AA genotypes	28.10	(78)	16.70	(27)	21.4	(15)	6.8	(3)	0.007	0.51 (0.31 to 0.84)	0.003	0.19 (0.06 to 0.62)
Bx genotypes	71.90	(200)	83.30	(135)	78.6	(55)	93.2	(41)	0.007	1.95 (1.19 to 3.18)	0.003	5.33 (1.6 to 17.72)
C4 linkage groups	33.50	(93)	35.80	(58)	35.7	(25)	27.3	(12)				
T4 linkage groups	21.60	(60)	37.00	(60)	32.9	(23)	45.5	(20)	0.0005	2.14 (1.39 to 3.28)	0.001	3.03 (1.57 to 5.85)
***KIR genes***												
**Group-A haplotype-associated KIR genes**												
*2DL1*	97.8	(272)	96.9	(157)	97.1	(68)	95.5	(42)				
*2DL3*	90.6	(252)	74.1	(120)	77.1	(54)	70.5	(31)	0.000003	0.29 (0.17 to 0.5)	0.0003	0.24 (0.11 to 0.52)
*3DL1*	95.7	(266)	90.1	(146)	91.4	(64)	88.6	(39)	0.02	0.41 (0.19 to 0.89		
*2DS4*	96.0	(267)	91.4	(148)	91.4	(64)	90.9	(40)	0.04	0.44 (0.19 to 0.98)		
**Group-B haplotype-associated KIR genes**												
*2DL2*	59.7	(166)	69.8	(113)	70.0	(49)	72.7	(32)	0.035	1.56 (1.03 to 2.34)		
*2DL5*	58.6	(163)	68.5	(111)	62.9	(44)	70.5	(31)	0.039	1.54 (1.02 to 2.3)		
*3DS1*	34.2	(95)	46.3	(75)	41.4	(29)	59.1	(26)	0.01	1.66 (1.112 to 2.47)	0.002	2.88 (1.45 to 5.33)
*2DS1*	36.3	(101)	55.6	(90)	44.3	(31)	70.5	(31)	0.0001	2.19 (1.48 to 3.25)	0.0001	4.18 (2.09 to 8.35)
*2DS2*	54.7	(152)	52.5	(85)	58.6	(41)	43.2	(19)				
*2DS3*	39.2	(109)	40.7	(66)	40.0	(28)	31.8	(14)				
*2DS5*	25.5	(71)	43.2	(70)	38.6	(27)	52.3	(23)	0.0001	2.22 (1.47 to 3.35)	0.0005	3.19 (1.67 to 6.12)
**Framework genes/pseudogenes**												
*2DL4*	100	(278)	100	(162)	100	(70)	100	(44)				
*3DL2*	100	(278)	100	(162)	100	(70)	100	(44)				
*3DL3*	100	(278)	100	(162)	100	(70)	100	(44)				
*2DP1*	97.5	(271)	88.3	(143)	88.6	(62)	86.4	(38)	0.0003	0.19 (0.08 to 0.47)	0.0019	0.16 (0.05 to 0.51)
*3DP1*	100	(278)	100	(162)	100	(70)	100	(44)				

Carrier frequency (%F) of each genotype is expressed as a percentage and defined as the number of individual having the genotype (N +) divided by the number of studied (n) in the study group.

### *B* haplotype-specific *KIR*s were increased in patients with breast cancer

Five of seven *B* haplotype-specific *KIR*s (*2DL2, 2DL5, 3DS1, 2DS1, 2DS5*) were significantly increased in patients with breast cancer than controls (Table 1). Three telomeric *B* haplotype-associated activating *KIR* genes, such as *3DS1, 2DS1,* and *2DS5,* were more prominently increased in patients with advanced-stage breast cancer (Table 1). Particularly, *KIR2DS1* was significantly at higher frequency in advanced-stage cancer patients compared to controls (70.5% vs. 36.3%, *p* = 0.0001, OR = 4.18, CI = 2.09–8.35). Inversely, two of four *A* haplotype-specific *KIR*s were decreased in patients with breast cancer compared to controls: *KIR2DL3* (74.1% vs. 90.6%, *p* = 0.000003, OR = 0.29, CI = 0.17–0.5), *KIR3DL1* (90.1% vs. 95.7%, *p* = 0.02, OR = 0.41, CI = 0.19–0.89). The *KIR2DL3* was further decreased in patients with advanced-stage breast cancer (70.5% vs. 90.6%, *p* = 0.0003, OR = 0.24, CI = 0.11–0.52).

### *KIR-HLA* gene combinations in patients with breast cancer and controls

No significant difference was observed in the frequencies of HLA-C1, C2, Bw4, and A3/11 ligands between breast cancer patients and controls (Suppl. Table 1). To examine whether *KIR-HLA* combinations confer risk for breast cancer, we compared the frequency of four well-characterized inhibitory *KIR* and cognate *HLA* class I ligand combinations (Table 2). No significant difference was found in the frequency of any inhibitory *KIR* and *HLA* class I ligand combinations between patients and controls. However, activating receptor and their putative *HLA* class I ligand combinations, such as *KIR2DS1* + *C2* + (52.4% vs. 26.9%, *p* = 0.001, OR = 2.98, CI = 1.54–5.79) and *KIR3DS1* + *Bw4* + (29.3% vs. 17.7%, *p* = 0.008, OR = 2.6, CI = 1.26–5.39) were increased in patients with advanced breast cancer compared to controls (Table 2).

**Table 2:** Carrier frequency of KIR+HLA class I ligand combinations in breast cancer patients and controls.

KIR- HLA combination	Healthy controls (n = 278)		Breast cancer						Comparison			
			All (n = 162)		Advanced (n = 44)		Early (n = 70)		All versus controls		Advanced versus controls	
									*p* value	OR (95% CI)	*p* value	OR (95% CI)
	%F	(N +)	%F	(N +)	%F	(N +)						
**Inhibitory KIR + HLA class I ligand**												
2DL1 + C2 +	74.7	(192/256)	67.9	(93/135)	79.5	(31/38)	57.9	(33/56)				
2DL2 + 2DL3 + C1 +	34.6	(92/256)	32.7	(50/140)	35.7	(15/38)	29.7	(19/56)				
3DL1 + Bw4 +	60.6	(143/233)	50.8	(62/116)	58.8	(20/32)	51.0	(26/48)				
3DL2 + A3/A11 +	40.0	(50/125)	42.4	(39/92)	31.8	(7/22)	37.8	(14/37)				
**Activating KIR + HLA class I ligand**												
2DS1 + C2 +	26.9	(73/256)	36.1	(53/135)	52.4	(22/38)	21.0	(13/56)			0.001	2.98 (1.54 to 5.79)
3DS1 + Bw4 +	17.7	(46/233)	25.9	(37/116)	29.3	(12/32)	18.6	(11/48)			0.008	2.6 (1.26 to 5.39)
**Inhibitory KIR + Activating KIR + HLA class I ligand**												
2DL1 + C2 + 2DS1 +	26.6	(72/256)	34.5	(51/135)	52.4	(22/38)	19.0	(2/56)			0.001	3.04 (1.56 to 5.89)
2DL1 + C2 + 2DS1 −	45.5	(120/256)	27.8	(42/135)	22.0	(9/38)	32.8	(21/56)	0.0004	0.46 (0.3 to 0.71)	0.005	0.34 (0.15 to 0.73)
3DL1 + Bw4 + 3DS1 +	15.6	(41/233)	20.1	(30/116)	29.3	(12/32)	14.5	(9/48)			0.033	2.23 (1.05 to 4.72)
3DL1 + Bw4 + 3DS1-	40.5	(102/233)	23.7	(32/116)	21.6	(8/32)	28.8	(17/48)	0.001	0.46 (0.28 to 0.73)	0.027	0.41 (0.18 to 0.92)
2DL1 + C2 + 3DL1 + Bw4 + 2DS1 + 3DS1 +	11.5	(30/233)	12.3	(19/116)	19.5	(8/32)	4.6	(3/48)				
2DL1 + C2 + 3DL1 + Bw4 + 2DS1 − 3DS1 −	25.9	(66/233)	12.8	(19/116)	12.5	(5/32)	16.1	(10/48)	0.002	0.42 (0.24 to 0.73)		

Coexistence of *KIR2DL1* + *C2* + , and activating counterpart *KIR2DS1* + was more frequent in patients with advanced breast cancer compared to controls (52.4% vs. 26.6%, *p* = 0.001, OR = 3.04, CI = 1.56–5.89) (Table 2, Fig. 3). In contrast, *KIR2DL1* + *C2* + without *KIR2DS1* is more frequent in controls compared patients (45.5% vs. 27.8%, *p* = 0.0004, OR = 0.46, CI = 0.3–0.71). The *KIR3DL1* + *Bw4* + , and its activating counterpart *KIR3DS1* + was more frequent in patients with advanced stage breast cancer compared to controls (29.3% vs. 15.6%, *p* = 0.033, OR = 2.23, CI = 1.05–4.72) (Table 2, Fig. 3). The *KIR3DL1* + *Bw4* + without activating counterpart *KIR3DS1* was more frequent in the controls compared to patients (40.5% vs. 23.7%, *p* = 0.001, OR = 0.46, CI = 0.28–0.73). The *KIR2DL1* + *C2* + plus *KIR3DL1* + *Bw4* + without their activating counterparts *KIR2DS1* and *3DS1* was more frequent in the controls compared to patients (25.9% vs. 12.8%, *p* = 0.002, OR = 0.42, CI = 0.24–0.73).

## Discussion

Aggregation of breast cancer in families indicates a predisposing genetic component for breast cancer risk^30^. Family studies using linkage analysis have identified several rare mutations with strong effects (i.e., highly penetrant), notably at *BRCA1, BRCA2, PALB2, ATM*, and *CHEK2* loci, conferring lifetime risk of breast cancer^31^. The large-scale genome-wide association studies (GWAS) have identified more than 200 susceptibility loci, each of which confers a small risk for breast cancer development^32^. However, the mechanism steering these genetic associations remains largely unknown because most variants are located in non-coding regions and are not in strong linkage disequilibrium with known protein‐coding variants^33^. Moreover, GWAS includes relatively few informative SNPs in the KIR region, and therefore analysis of the KIR region has been impractical because its extraordinary structural diversity leaves few locations suitable for designing binary SNP markers^34^. Therefore, much of the KIR genetic contribution to breast cancer risk remains unknown.

NK cell surveillance is an essential activity in defending tumor initiation and metastasis^35^. According to the “missing-self” hypothesis, NK cells complement T cell immunity by killing cancer cells that downregulate MHC class I molecules to escape class I-restricted T cell response^36^. Presumably, defects in NK cell number and activity play a role in breast cancer initiation and progression. Consistent with this notion, a substantial reduction of blood NK cell cytotoxicity in women with breast cancer, particularly in women with advanced-stage breast cancer, was noted compared to healthy individuals^9,10^. Individuals with high incidences of familial breast cancer exhibit significantly reduced NK cell cytotoxicity in peripheral blood^37^. The advanced breast cancer patients have an increased proportion of more immature and less cytotoxic CD56^bright^CD16^+/−^ NK cell subset in their peripheral blood, which might account for at least part of the reduced levels of cytotoxic functions observed in these patients^38^. The molecular mechanism underlying the impaired NK cell cytotoxicity and antitumor effect in breast cancer is not identified.

The interaction of inhibitory KIR with specific cognate HLA class I ligand makes NK cells matured to acquire full effector function, developmental programming termed “licensing”^16,17^. In the absence of inhibitory KIR-HLA interactions, NK cells became hyporesponsive or anergic. The distribution of four inhibitory KIR-HLA class I ligand combinations is comparable between breast cancer patients and controls, indicating that the development of functionally active NK cells in patients might be similar to those of controls. However, NK licensing is not entirely permanent, and the functional activity of mature NK cells can be reset by new HLA environment in tumor tissue with reduced HLA class I expression^39^, a mechanism that tumor develops to evade from adoptive immune response^40^.

The activating KIR-based HLA class I-dependent licensing may also influence NK cell unresponsiveness to transformed cells. Although the mechanisms have not yet been identified, education by activating KIRs shares features with the hyporesponsiveness induced by chronic stimulation of other activating receptors expressed by NK cells. For example, chronic exposure to NKG2D ligands in mice renders NK cells hyporesponsive to target cells^41^. A recent study found that the expression of NKG2D on blood NK cells was higher in breast cancer patients than the levels documented in healthy females^10^. Similarly, when the ligand (m157) for the activating Ly49H is constitutively expressed, mouse Ly49H + NK cells become hyporesponsive^42^. NK cells expressing activating KIR2DS1 are hyporesponsive in the presence of self-HLA-C2 ligands^43^ and thus unable to mount an efficient response against breast cancer. This disarming model of licensing emphasizes the crucial role of activating KIRs 2DS1 and 3DS1 and interactions with their HLA class I ligands in developing anergic NK cells (disarmed), which are hyporesponsive and are not able to defend against the tumor. All patients with advanced cancer carrying 2DS1 + C2 or 3DS1 + Bw4 also had their inhibitory KIR counterparts 2DL1 and 3DL1, respectively. In contrast, the 2DL1 + C2 and 3DL1 + Bw4 pairs without their activating KIR counterparts were significantly higher in controls than patients. These data suggest that NK cells expressing inhibitory KIR to the cognate HLA class I ligands in the absence of putative activating KIR counterpart are instrumental in antitumor response.

It also remains possible that activating KIR receptors could recognize altered HLA class I complexes, e.g., specific HLA/peptide complexes^44^. It is possible that the activating KIRs could directly bind neoantigens explicitly expressed on breast cancer cells, which may suppress cytolytic function but trigger cytokine release. Supporting this possibility, KIR2DS4 has been suggested binding to an unidentified protein expressed on melanoma-derived tumor cells, independently of HLA class I^45^. Particularly, KIR2DS1 has been shown to displays a certain degree of peptide selectivity in its binding to HLA class I^46^, indicating that the functional outcome of activating KIRs can be modulated by the nature of the presented peptide.

The human *KIR* genotypes can be simply divided into two groups, *AA* and *Bx*, with quantitively and qualitatively contrasting *KIR* gene content^15^. We found a striking association of *Bx* genotypes with breast cancer. Three of four prior studies suggested an association between breast cancer and *B*-haplotype-specific *KIR* genes^47–50^. Consistent with our findings, Jobim et al*.* found a strong association between KIR2DL2 and Brazilian women with breast cancer^48^. However, this study could not find any association with other KIR genes, such as KIR2DS2, 2DS3, and 2DL5, which are located at close proximity to 2DL2 with strong linkage disequilibrium. Jobim et al*.* also reported an association between KIR2DL2 + C1 in breast cancer, which was not observed in our study. Oztruk et al*.* reported a strong association between KIR2DS1 and patients with breast cancer in Turkey^49^, which is in agreement with our findings. However, they could not find an association with KIR2DS1-linked genes, such as 3DS1 and 2DS5.

Using a new cohort of breast cancer patients and controls from the Fars province, our collaborator Prof. Abbas Ghaderi and team recently reported an association between Iranian breast cancer patients and Bx KIR genotypes, centromeric Bx genotypes, and B-haplotypes carrying C4T4 motif (positive for seven KIRs: 2DL2, 2DS2, 2DS3, 2DL5, 3DL1, 2DS1, and 2DS3)^50^, which is in agreement with our findings. However, the study confirmed the association of individual B-haplotype-associated KIR genes only with breast cancers expressing estrogen receptors. Breast cancer positive for progesterone receptor or human epidermal growth factor 2 (HER2) were not associated with B-haplotype-specific KIRs. Moreover, the HLA class I ligands were not analyzed in this study. In total contradiction to our findings, Alomar et al*.* reported a significant decrease in the frequencies of KIR2DS2, 2DS3, and Bx genotypes in 50 Saudi women with breast cancer compared to 65 controls^47^. HLA ligands were analyzed by only Turkish and Saudi studies. Inconsistent results observed between the studies are presumably contributed by multiple factors, including ethnic and population disparity in KIR and HLA genome, the differential composition of histologic breast cancer phenotypes, and small sample sizes.

The *B*-haplotype *KIR*s were correlated with an increased risk of other solid and hematological malignancies, including leukemia^51^, cervical neoplasia^52^, Hodgkin lymphoma^53^, gastric cancer^54^, head and neck squamous cell carcinoma^55^, urothelial bladder cancer^56^, colorectal adenocarcinoma^57^, systemic sclerosis^58^, and meningioma^59^. The *B* haplotype-specific *KIR*s, particularly those located at the telomeric half (*3DS1, 2DS1,* and *2DS5*), were observed to be prominently increased in patients with an advanced stage of breast cancer. These results contrast with the classical view that activating NK cell receptors mediate spontaneous lysis of transformed cells and protect against the tumor^60^.

Given our study's retrospective design, further investigations are warranted in a prospectively accrued patient population to substantiate our findings. The number of patients included in our study was insufficient to evaluate the impact of *KIR-HLA* combinations in tumors with different phenotypes, such as estrogen receptor-positive (ER +), progesterone receptor-positive (PgR +), and HER2 + . Therefore, further systematic studies should be focused on determining the impact of combined KIR + HLA combinations using multivariate analysis. The limitation of our KIR-binding HLA epitope typing is its inability to discriminate HLA allotypes (e.g., Cw*05:01, Cw*02:02), which can differ in binding affinity^61^. In summary, our results provide a genetic basis for impaired NK cell antitumor activity in breast cancer. The *KIR-HLA* associations observed in this study provide further insight into genetic susceptibility to breast cancer, improving the utility of genetic risk scores for individualized screening and follow-up recommendations for earlier implementation of breast cancer risk-reduction strategies. Moreover, our results suggest that autologous activated NK cell clones with select KIR-HLA composition favoring antitumor activity could be a promising immunotherapeutic strategy against breast cancer.

## Materials and methods

### Study subjects and samples

A cohort of 162 women with breast cancer and 278 healthy controls from the southern part of Iran (Fars province) were included in this study. The patients were recruited at Faghih hospital, Shiraz University of Medical Sciences. The age-matched controls were collected from the same geographical area. The clinical and pathological characteristics were collected from patient medical records. Table 3 shows the distribution of clinicopathological characteristics of breast cancer. The breast cancer patients were categorized according to TNM staging^62^, and grouped as either early stage (0, I, and II) or advanced stage (III and IV) of disease. The study was reviewed and approved by the Medical Research Ethics Committee of Shiraz University of Medical Sciences and UCLA Institutional Review Board of human research protection. Genomic DNA was extracted from peripheral blood samples using either the standard salting-out method or by QIAamp blood kit (Qiagen, Hilden, Germany). The quality and quantity of DNA were determined by UV spectrophotometry, and the concentration was adjusted to 100 ng/μL. All DNA samples received at UCLA were de-identified and only marked as having been obtained from patients with breast cancer or controls. Informed consent was obtained from all subjects. Data obtained were Health Insurance Portability and Accountability Act (HIPAA) compliant, and the study adhered to the tenets of the Declaration of Helsinki. All methods were carried out in accordance with relevant guidelines and regulations.

**Table 3:** Clinicopathological characterestics of patients with breast cancer.

	n = 162	
	%F	(N +)
**Tumor type**		
Infiltrative ductal carcinoma (IDC)	68.5	(111)
Medullary carcinoma	3.7	(6)
Metaplastic carcinoma	2.5	(4)
Mucinous carcinoma	0.6	(1)
Lobular carcinoma	2.5	(4)
Phyllodes tumor	1.9	(3)
Ductal carcinoma in situ (DCIC)	1.2	(2)
Unknown	19.1	(31)
**Tumor side**		
Left	37	(60)
Right	34.6	(56)
Bilateral	1.2	(2)
Unknown	27.2	(44)
**Histological tumor grade**		
I—Well differentiated	14.8	(24)
II—Moderately differentiated	41.4	(67)
III—Poorly differentiated	9.3	(15)
Unknown	34.6	(56)
**Lymph node involvement**		
Positive	44.4	(72)
Negative	30.9	(50)
Unknown	24.7	(40)
**TNM staging**		
I	6.8	(11)
II	36.4	(59)
III	25.9	(42)
IV	1.2	(2)
Unknown	29.6	(48)

### *KIR* genotyping and genotype/haplogroup classification

The presence and absence of 16 *KIR* genes were determined using our previously developed duplex SSP-PCR typing method^63^. Ambiguous and unusual *KIR* genotypes were resolved by using the alternative SSP-PCR typing method^27^. Based on the presence and absence of *KIR* genes, we divided the study subjects into two groups: the *AA* and *Bx* genotype carriers. The *AA* genotype subjects carried only *KIR3DL3-2DL3-2DL1-2DP1-3DP1-2DL4-3DL1-2DS4-3DL2* genes that are characteristic of *A*-haplotype. The rest were regarded as *Bx* genotype carriers (*AB* heterozygous and *BB* homozygous carriers). Based on our previous linkage disequilibrium analyses, we determined the frequency of *B*-haplotype-specific *KIR* gene clusters^64,65^. One of them comprises *KIR2DS2-2DL2-2DS3-2DL5B* genes and is located at the centromeric half of the *KIR* gene complex (termed *C4* linkage group). In contrast, another cluster contains *KIR3DS1-2DL5A-2DS5-2DS1* genes and is located at the telomeric half of the complex (termed *T4* linkage group).

### *HLA* class I ligand typing by novel direct sequencing

We developed a novel direct DNA sequencing method to determine KIR-binding HLA-A, -B, and -C ligands. The procedure starts with gene-specific amplification of exon 2 and 3 of *HLA-A, -B*, and -*C* loci followed by direct sequencing of PCR amplicons (Suppl. Fig. 1, Suppl. Table 2). The primers amplify all common and well-documented HLA-A, -B, and -C alleles^66^. However, the HLA-B amplification excludes HLA-B*73:01. The reverse primers used in the HLA-B specific amplification (3BIn3-37R) binds to the intron-3 region from nucleotide 1028 to 1050. Since the HLA-B*73:01 allele has mutations at reverse primer annealing site at nucleotide 1032 from G to A and 1038 from G to C, the HLA-B*73:01 is not be amplified by HLA-B PCR. However, HLA-B*73:01 is amplified by HLA-C specific amplification. Gene-specific PCR reaction (20 μL volume) comprised a final concentration of 1 × LT buffer II, 500 μM of each deoxyribonucleotide triphosphates (dNTPs), 0.3 μM of each forward and reverse primers to either *HLA-A, -B* or -*C*, 1.5 U of LT Tgo DNA polymerase (Roche Applied Science, Germany), and 100 ng genomic DNA. The PCR thermal cycling was performed in ABI 9700 GeneAmp PCR system (Applied Biosystems, USA) using the following thermal cycles: initial denaturation for 1 min at 94 °C; 12 cycles at 94 °C for 10 s, and 68 °C for 2.5 min; 20 cycles of 94 °C for 15 s, 63 °C for 30 s, and 68 °C for 2 min; and a final extension at 68 °C for 7 min. The PCR products (2 μL) were subjected to electrophoresis on 2% agarose gel to visualize specific bands with the expected size.

The PCR amplicons were purified from unincorporated primers and dNTPs by digesting with ExoSAP-IT exonuclease-I (USB Corporation, Cleveland, OH) according to the manufacturer's protocol and were used as a template in the sequencing reactions. Then the segments of exon 2 that encode the KIR-ligands were sequenced at both directions using the BigDye terminator V1.1 cycle sequencing kit (Applied Biosystems, Foster City, CA). Sequencing reactions (10 μL) comprise 2 μL of sequencing reagent premix, 1 μL dilution buffer, 0.3 μL of sequencing primer (10 pM/μL), and 2 μL of purified PCR amplicon. The following PCR thermal cycling profile was used: 25 cycles of 96 °C for 20 s, 53 °C for 20 s, 60 °C for 1 min, and soak at 4 °C. Once the cycling was completed, the sequencing reactions were precipitated using sodium acetate/EDTA buffer and ethanol to concentrate the reactions and to eliminate unincorporated fluorescent-labeled nucleotides. The precipitates were resuspended in 15 µL of Hi-Di deionized formamide (Applied Biosystems), denatured by heating at 95 °C for 2 min, and loaded into the ABI PRISIM_TM_ 310 capillary sequencer (Applied Biosystems). Finally, sequence analysis was performed using Assign SBT v3.5.1 software (Conexio Genomics, Western Australia), which can combine both forward and reverse sequences files to inspect and edit the electropherograms. The Assign program assigned the alleles by comparing the test sequences with a library of known *HLA-A, -B*, and -*C* sequences downloaded from the international ImMunoGeneTics (IMGT-HLA Database (http://www.ebi.ac.uk/imgt/hla). The KIR-binding HLA class I ligands were deduced from the assigned alleles. We have validated this method by using a panel of 31 UCLA DNA standards that includes most core HLA class I types (Suppl. Table 3).

### Data analysis and statistical methods

The percentage of each *KIR* gene in control and patient groups was determined by direct counting (individuals positive for the gene divided by individuals tested per population × 100). Differences between the study groups in the distribution of each *KIR* genotypes, *KIR* genes, *HLA* ligands, and *KIR-HLA* combinations were estimated by the two-tailed Fisher Exact probability (P) test, and *p* < 0.05 was considered to be statistically significant. Odds ratio (OR) and 95% Confidence Intervals (CI) were calculated to determine the magnitude and statistical significance of associations^67^.

## Supplementary Information

Supplementary Information

## Data Availability

The datasets generated and analyzed during the current study are available from the corresponding author on reasonable request.
