# Supplementary material for: Coexistence of inhibitory and activating killer-cell immunoglobulin-like receptors to the same cognate HLA-C2 and Bw4 ligands confer breast cancer risk
Source: Sci Rep. 2021 Apr 12;11:7932. doi: 10.1038/s41598-021-86964-y (PMC8041876; doi:10.1038/s41598-021-86964-y)
Supplement: Supplementary file 1 — Supplementary Information [file 41598_2021_86964_MOESM1_ESM.pdf]

Coexistence of inhibitory and activating killer-cell immunoglobulin-like receptors to the same cognate HLA-C2 and Bw4 ligands confer breast cancer risk

Elham Ashouri, Karan Rajalingam, Shaghik Barani, Shirin Farjadian, Abbas Ghaderi, Raja Rajalingam.

____

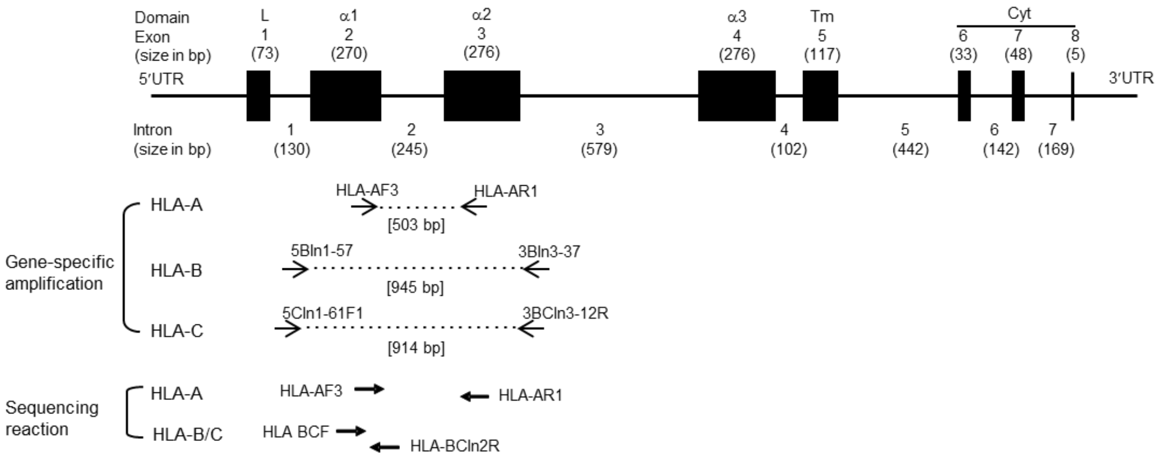

Suppl. Figure 1. **Schematic representation of the strategy used for KIR-binding HLA class I epitope typing.** The exon (dark boxes) and intron (line connecting the dark boxes) organization of the HLA class I genes is depicted at the top. The size of each exon and intron determined on the basis of the HLA-A*01:01 sequence is provided in parenthesis. Arrows show the annealing location and direction of the primers used for gene-specific amplification and sequencing reaction. The nucleotide sequences of these primers are listed in the S1 Table.

Suppl. Table 1. Carrier frequency of HLA class I ligands in breast cancer patients and controls.

HLA	Healthy controls		Breast cancer					
			All (n=162)		Early (n=70)		Advanced (n=44)	
	%F	(N+)	%F	(N+)	%F	(N+)	%F	(N+)
HLA-C1	72.7	(186/256)	76.3	(103/135)	75	(42/56)	71.1	(27/38)
HLA-C2	76.2	(195/256)	70.4	(95/135)	60.7	(34/56)	81.6	(31/38)
HLA-Bw4	63.9	(149/233)	59.5	(69/116)	58.3	(28/48)	68.8	(22/32)
$Bw4^{I80}$	54.1	(126/233)	52.6	(61/116)	56.2	(27/48)	59.4	(19/32)
$Bw4^{T80}$	17.2	(40/233)	9.5	(11/116)	6.2	(3/48)	12.5	(4/32)
HLA-A3/A1	40.0	(50/125)	40.4	(36/89)	37.8	(14/37)	31.8	(7/22)

Carrier frequency (%F) of each HLA gene/genotype is expressed as percentage and defined as the number of individual carrying the gene/genotype (N+) divided by the number of subjects studied (n) in the group.

Suppl. Table 2. Oligonucleotide primers used to characterize the KIR-binding HLA class I ligands.

Gene	Direction	Name	Sequence (5'-----3')
Primers for gene-specific PCR amplification			
HLA-A	Forward:	HLA-AF3	GACAGCGACGCCGCGAGCC
	Reverse:	HLA-AR1	CGCCGCGGTCCAAGAGCG
HLA-B	Forward:	5BIn1-57	GGGAGGAGMGAGGGGACCSCAG
	Reverse:	3BIn3-37R	GGAGGCCATCCCCGGCGACCTAT
HLA-C	Forward:	5CIn1-61F1	CGAGGKGCCCKCCCGGCGA
	Reverse:	3BCIn3-12R	GGAGATRGGGAAGGCTCCCCACT
Primer for sequencing			
HLA-A	Forward:	HLA-AF3	GACAGCGACGCCGCGAGCC
	Reverse:	HLA-AR1	CGCCGCGGTCCAAGAGCG
HLA-B & C	Forward:	HLA-BCF	GACAGCGACGCCGCGAGTCC
	Reverse:	HLA-BCIn2R	GACCCSGGCCGTMCGTSGG

Suppl. Table 3. List of DNA standard used to validate HLA class I ligand typing.

Sample #	HLA-A allele	KIR ligand	Sample #	HLA-B allele	KIR ligand	Sample #	HLA-C allele	KIR ligand
126	02:01, 80:01	-, -	126	14:01, 44:02	_, Bw4	137	03:04, 06:02	C1, C2
137	11:01, 30:01	A11, -	137	13:01, 40:01	Bw4, _	155	05:01, 06:02	C2, C2
144	01:01, 69:01	-, -	155	37:01, 44:02	Bw4, Bw4	169	03:04, 07:02	C1, C1
169	01:01, 02:01	-, -	169	07:02, 40:01	_, _	210	01:02, 01:02	C1, C1
210	02:06, 02:06	-, -	210	55:02, 56:01	_, _	259	30:42, 06:02	C1, C2
227	11:01, 11:02	A11, A11	259	15:10, 57:01	_, Bw4	271	01:02, 14:02	C1, C1
242	02:03, 11:01	-, A11	271	46:01, 51:01	_, Bw4	272	03:04, 07:01	C1, C1
249	11:02, 30:01	A11, -	272	18:01, 40:02	_, _	281	04:01, 07:01	C2, C1
259	23:01, 31:01	Aw4, -	281	08:01, 35:01	_, _	322	03:02, 04:01	C1, C2
260	36:01, 68:01	-, -	322	53:01, 58:01	Bw4, Bw4	389	08:02, 12:03	C1, C1
271	02:01, 02:03	-, -	389	14:02, 35:03	_, _	390	04:01, 05:01	C2, C2
272	02:01, 31:01	-, -	390	18:01, 35:02	_, _	211	07:02, 14:02	C1, C1
283	01:01, 32:01	-, Aw4	284	35:17, 44:03	_, Bw4	233	07:02, 18:01	C1, C2
322	23:01, 66:02	Aw4, -	386	27:05, 40:01	Bw4, _	251	04:01, 15:02	C2, C2
400	02:01, 30:01	-, -	231	37:01, 58:01	Bw4, Bw4	284	04:01, 16:01	C2, C1
430	01:01, 24:02	-, Aw4	452	44:02, 52:01	Bw4, Bw4	310	01:02, 07:01	C1, C1
431	30:04, 33:03	-, -	144	51:01, 73:01	Bw4, C1	342	02:02, 16:01	C2, C1
438	03:02, 68:02	A3, -	431	14:01, 44:03	_, Bw4	361	02:02, 17:01	C2, C2
441	68:02, 74:01	-, -	438	15:17, 49:01	Bw4, Bw4	379	02:02, 18:01	C2, C2
444	01:01, 26:01	-, -	227	15:02, 27:04	_, Bw4	380	03:05, 16:01	C1, C1
449	02:01, 24:02	-, Aw4	249	13:02, 27:04	Bw4, Bw4	382	07:02, 07:02	C1, C1
452	03:01, 24:02	A3, Aw4	242	27:04, 40:01	Bw4, _	386	01:02, 03:04	C1, C1
387	02:01, 24:02	-, Aw4	192	51:01, 73:01	Bw4, C1	391	07:02, 08:01	C1, C1
284	01:01, 24:02	-, Aw4	246	27:05, 46:01	Bw4, _	400	03:04, 17:01	C1, C2
310	11:01, 32:01	A11, Aw4	441	53:01, 58:02	Bw4, Bw4	430	07:02, 12:02	C1, C1
379	01:01, 74:01	-, -	276	44:03, 57:01	Bw4, Bw4	431	08:02, 14:03	C1, C1
380	11:01, 24:02	A11, Aw4	176	07:02, 35:08	_, _	437	04:03, 12:03	C2, C1
382	24:02, 24:07	Aw4, Aw4	173	44:02, 44:02	Bw4, Bw4	438	05:01, 05:01	C2, C2
386	02:01, 66:01	-, -	156	27:04, 39:01	Bw4, _	443	08:02, 18:01	C1, C2
388	24:07, 24:10	Aw4, Aw4	437	13:01, 40:10	Bw4, _	444	06:02, 15:02	C2, C2
391	02:07, 11:01	-, A11	196	27:05, 44:02	Bw4, Bw4	449	07:04, 15:02	C1, C2
